# Finite Element Analysis of Densification Process in High Velocity Compaction of Iron-Based Powder

**DOI:** 10.3390/ma17133085

**Published:** 2024-06-23

**Authors:** Miao Liu, Yan Cao, Chaorui Nie, Zhen Wang, Yinhuan Zhang

**Affiliations:** 1 School of Mechatronic Engineering, Xi’an Technological University, Xi’an 710021, China; liumiao@st.xatu.edu.cn (M.L.); zhangyinhuan@st.xatu.edu.cn (Y.Z.); 2 School of Automotive Engineering and General Aviation, Shaanxi Vocational and Technical College, Xi’an 710100, China; niechaoruiyaofei@163.com; 3 School of Mechanical Engineering, Xijing University, Xi’an 710123, China; zhen.wang.xian@gmail.com

**Keywords:** densification process, compaction equation, high velocity compaction

## Abstract

A finite element model based on elastic–plastic theory was conducted to study the densification process of iron-based powder metallurgy during high velocity compaction (HVC). The densification process of HVC at different heights was simulated using MSC Marc 2020 software with the Shima–Oyane model, and compared with the experimental results. The numerical simulation results were consistent with the experimental results, proving the reliability of the finite element model. Through finite element analysis and theoretical calculation, the high-speed impact molding process of metal powder was analyzed, and the optimal empirical compaction equation for iron-based powder high-speed impact molding was obtained. At the same time, the influence of impact velocity and impact energy on the relative density distribution cloud map and numerical values of the compact was analyzed.

## 1. Introduction

**Figure 1:**
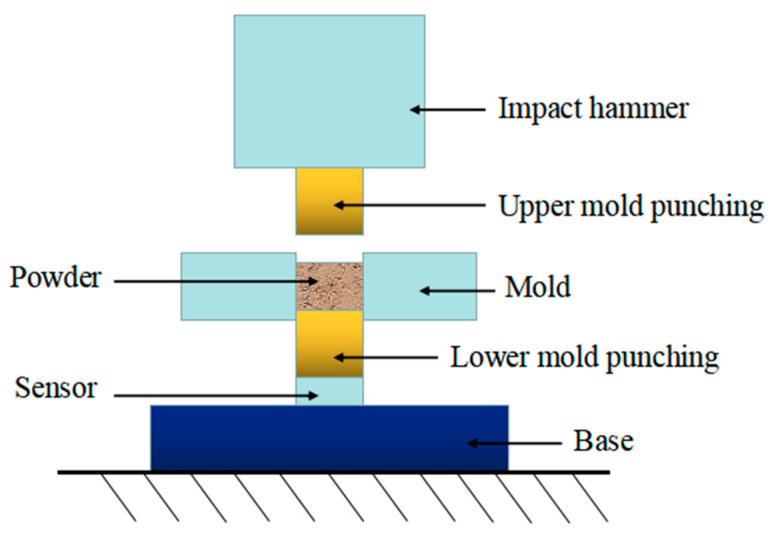
Experiment device schematic diagram of HVC.

High velocity compression (HVC) is a rapid prototyping process first proposed by Paul Skoglund at the 2001 American Metal Powder Federation to improve densification [1]. The basic principle of the process is as follows: the mixed powder is fed into the mold for high-speed impact (2–30 m/s) compression molding, and then the compact is pushed out and transferred to the sintering process. One compression can be completed within 0.02 s, and no more than five high-speed impact compressions can be achieved within a time interval of 0.30–1.00 s. High energy accumulation can be achieved through repeated compression, thereby improving the comprehensive mechanical properties of the prepared material. The simplified principle diagram is shown in Figure 1. HVC has the advantages of high production efficiency, low preparation cost, high and uniform density of the obtained compacts, and can be combined with warm pressing technology, mold wall lubrication technology, etc., to prepare composite material compacts with high density and low elastic after-effects, such as various metals and ceramics. At present, HVC has achieved the production of single-layer parts such as cylinders, rods, rings, and cams, as well as complex parts such as bearing caps and tooth caps [2], and is widely used in the preparation of parts in fields such as automobiles and aerospace. Therefore, the study of pressing process and the influencing factors of HVC is important in our application of powder metallurgy.

Numerical simulation has the advantages of a short cycle, high efficiency, and the ability to reproduce the pressing process. It is widely used in the research of the HVC molding densification process of metal powders. Yu Shiwei et al. [3] used the Drucker Prager Cap model and the transient explicit dynamic finite element method to simulate the HVC process with ABAQUS 2014 software, and obtained the density changes and distribution patterns of the powder during the pressing process. Quantitative analysis was conducted on the effects of wall friction coefficient, heigh-to-diameter ratio, unit mass energy, and initial loose density on the density size and uniformity. Based on the plastic deformation theory of powder materials and the Johnson–Cook constitutive equation, Wang Yan dong [4] established a simplified two-dimensional disk model of powder core-shell elements, and studied the effects of the initial packing structure, impact velocity, pressing pressure, and shell thickness (Al content) on the densification and temperature rise of compacts. Zhou Jian et al. [5] established a three-dimensional small ball particle model using the discrete element method to simulate the uniaxial high-speed pressing process of titanium alloy powder, and studied the effects of unit mass impact energy, friction coefficient, etc., on the final compact density and compaction performance. Li Lei [6] established a two-dimensional finite element model of the HVC process of metal powder based on the theory of continuum mechanics and the Shima–Oyane yield criterion. The study analyzed the effects of pressing speed, wall friction coefficient, pressing method, and aspect ratio on the density, distribution uniformity, and other performance indicators of the compact. An Xi zhong et al. [7] established a three-dimensional modeling of aluminum powder based on the Shima–Oyane model and simulated the powder pressing process. The research has found that the radial flow direction of particles shows the opposite distribution in the upper and lower regions of the compact. As the unloading process progresses, the elastic recovery of the green billet gradually increases. At the same time, it has been found that using external force cyclic loading can improve the overall density and distribution uniformity of the green billet. Wang Wen chao [8] adopted the MSC Marc software based on continuum mechanics with a modified Shima–Oyane model to establish a three-dimensional continuum powder model, simulating the biaxial molding process of macro-scale Cu-Al composite powder materials. The effects of initial packing density, friction coefficient, and holding time on the relative density and uniformity of the compact were studied.

In summary, the current numerical simulation methods for powder metallurgy forming mainly include the macro-scale continuum mechanics finite element method, the micro-scale discrete element method, and the coupled multi-particle finite element method. The discrete element method and multi-particle finite element method are mostly used to model two-dimensional disk models or three-dimensional spherical particles, and the scale of a single particle is enlarged. The total number of particles is much smaller than the actual situation, which is to study the local amplification, and which is inconsistent with the actual situation, resulting in a large error in the quantified relative density of compression. If the number of particles increases, the computational workload will be very large and the computational efficiency will be low. The discrete element method and multi-particle finite element method are suitable for studying the movement direction, velocity, and interaction relationship of particles at the micro-scale, but not for studying the compression equation at the macro-scale and the density distribution cloud map of the compact.

The elastic–plastic finite element method based on continuous media can accurately analyze the macroscopic overall deformation and densification degree of powder during the compression molding process. It describes its deformation behavior through the constitutive model of the system as a whole. It is assumed that the powder particles are a continuous medium, established using the theory of plastic mechanics, and formed by pressing at room temperature. The mechanical properties of the material do not change with time. By organizing and analyzing the relevant literature, the modeling theory research of scholars is divided into the following five categories: the ellipsoidal yield criterion model, consistent constitutive model, soil yield criterion, plastic intrinsic time theory, and rheological theory. The ellipsoidal yield model refers to a model represented by the modified models studied by scholars such as Kuhn-Downey [9], Green [10], Shima–Oyane [11], etc., which presents an ellipsoidal shape in space.

The macroscopic elastic–plastic ellipsoid model can effectively simulate the HVC process of powders, quantitatively analyze the densification process, and analyze the impact of various influencing factors on the performance of the compacts. Among them, the Shima–Oyane model is currently the main method applied in research. Therefore, this paper adopts the continuum mechanics finite element method to establish a three-dimensional macroscopic model of powder particles, to analyze the densification process of metal powder HVC process, empirical formulas of compression equations, and the influence of impact speed and impact energy on the relative density of the compact.

## 2. Materials and Methods

This paper chose the MSC Marc software which conducts finite element simulations of the HVC process of metal powders. It not only has the Shima–Oyane model installed that can accurately describe powder metallurgy materials, but also effectively calculates nonlinear large deformation problems. There is also a transient dynamics module specifically suitable for HVC simulation.

### 2.1. Material Properties

The relationship characteristic model for the HVC process of iron-based powder was established in MSC Marc, the powder material was an elastic–plastic body, and the relevant material properties were defined by inputting the relationship curve between the elastic modulus, Poisson’s ratio, and the density of the material.

The powder matrix material was pure iron, and the entire deformation process was considered to be a strain problem of isotropic elastic–plastic materials. The empirical formula for flow stress–strain is as follows [6]:

(1) $$\sigma =210\times \left(1+3.2857\varepsilon^{0.6}\right)$$

The core of the plasticity theory of metal powders with porous characteristics was the yield criterion, which refers to the principle that a certain point in the powder body enters plasticity from an elastic state and remains in a plastic state under different loads. This paper adopted a modified Shima–Oyane yield criterion model to analyze the flow pattern and relative density distribution of powder metal. The yield criterion model [12,13] of the material is provided in the following:

(2) $$F=\frac{1}{\gamma }{\left(\frac{3}{2}\sigma^{d}\sigma^{d}+\frac{p^{2}}{\beta^{2}}\right)}^{\frac{1}{2}}-\sigma_{y}$$

where $\sigma_{y}$ is uniaxial yield stress;$\sigma^{d}$ is deviatoric stress component tensor; and *p* is the hydrostatic pressure. γ and β are material parameters related to relative density, and their power-law model expression is as follows:

(3) $$\gamma ={\left(q_{1}+q_{2}\rho^{q_{3}}\right)}^{q_{4}}$$

(4) $$\beta ={\left(b_{1}+b_{2}\rho^{b_{3}}\right)}^{b_{4}}$$

where ρ is relative density, when the powder is compacted and the relative density reaches 1, the above equation becomes the von Mises yield criterion for dense bodies, and the Poisson’s ratio reaches 1. $q_{1}$, $q_{2}$, $q_{3}$, $q_{4}$, $b_{1}$, $b_{2}$, $b_{3}$, and $b_{4}$ are the parameters that determined γ and β, obtained through uniaxial compression and triaxial compression tests.

Shima et al. [11,13] determined the relevant parameter values through the compression molding experiment of copper powder and proved its applicability to iron powder and aluminum powder. Among them, $q_{1}=0,\;q_{2}=1,\;q_{3}=1,\;q_{4}=2.5,\;b_{1}=5.9,\;b_{2}=-5.9,\;b_{3}=1,\;and\;b_{4}=-0.514$. By substituting them into Formulas (3) and (4), the following can be obtained [14]:

(5) $$\gamma =\rho^{2.5}$$

(6) $$\beta ={\left(5.9-5.9\rho \right)}^{-0.514}$$

The elastic modulus refers to the slope of a straight line in the stress–strain curve relationship of a material during the elastic deformation stage, which characterizes the strength of the bonding between atoms in the lattice. It is defined by the intrinsic qualities of the material itself and is not influenced by shape features. In the HVC process of metal powder, the elastic modulus will change with the continuous compression in volume and the increase in density, which can be expressed as a function of the relationship between the elastic modulus *E* and the relative density ρ. The relationship between the elastic modulus and density of the powder is as follows:

(7) $$E=E_{c}\rho^{3.4}$$

Poisson’s ratio is an important parameter that directly reflects the lateral flow of metal powder during compression, and has a significant impact on its numerical simulation results. There are three types of Poisson’s ratio models suitable for metal powder compression molding: Kuhn Poisson’s ratio model, Poisson’s ratio normal model, and the tongue line function model [6,15]. The tongue line function model incorporates the advantages of the Kuhn model and the normal model, and can accurately describe the Poisson’s ratio relationship characteristics of materials regardless of whether the relative density is high or low. Therefore, this paper chose the tongue line function model as the Poisson’s ratio model, and its expression is as follows:

(8) $$ʋ=\frac{0.03}{\left(1-\rho^{2}\right)+0.06}$$

### 2.2. Analysis of Contact and Friction Relationship

The contact during the HVC process includes the contact between the particles and the contact between the particles and the mold wall. These contact states constantly change over time and exhibit nonlinear characteristics. The state between particles is determined by the distance tolerance value between unit nodes. If the distance between nodes is greater than the tolerance setting value, it is considered as a separated state. If the distance between nodes is less than the tolerance setting value, it is considered as a contact state. This is achieved through the contact function included in the MSC Marc software which enables contact detection and constraint determination of particle units. These are achieved through the touch functions included in the MSC Marc software. In order to ensure that the particle volume remains constant during the powder pressing process, it is assumed that two particles in contact with each other have no penetration phenomenon [6,16].

There are three types of contact bodies in MSC Marc, namely, deformable contact bodies, rigid contact bodies, and symmetric contact bodies. In order to improve computational efficiency and simulation reliability, this paper set the powder as a deformable contact body, and set the inner mold wall, heavy hammer, and upper and lower mold punches as rigid contact bodies. The contact table is defined as automatic full contact. The paper selected the Coulomb arctangent and Nodal stress model option for the touch type. At the same time, the large strain option was selected in the nonlinear method, and the updated Lagrangian method was selected for the deformation superposition process.

The modified Coulomb friction criterion was used, assuming that the friction coefficients between the powder and the inner surface of the grinding tool and the powder, and between the powder and the upper and lower die punches, were equivalent.

### 2.3. FEA Model, Initial Conditions and Constraints

**Figure 2:**
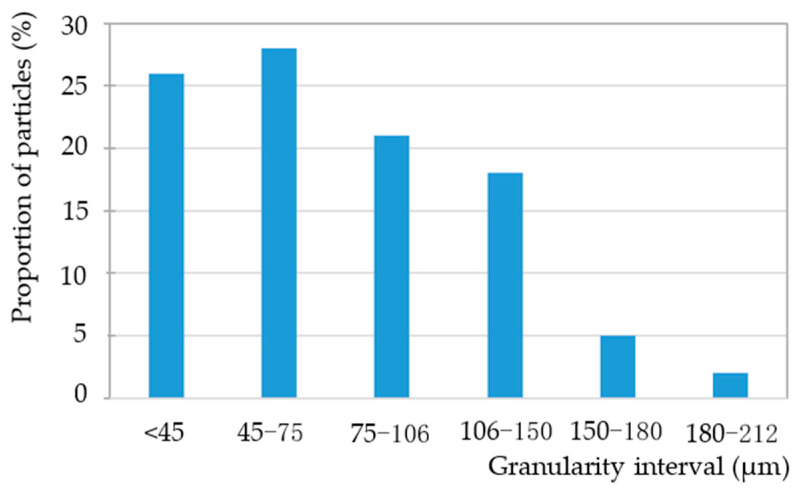
Particle size distribution of the powder.

**Figure 3:**
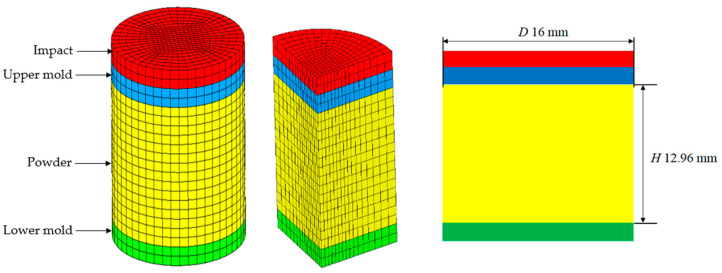
Simplified finite element model of HVC process.

The object of simulation was Distaloy AE iron-based powder with a density of 7.86 g/cm^3^, with its composition and particle size distribution as shown in Table 1 and Figure 2. The HVC model and dimensions of specimen are shown in Figure 3 and Table 2. The falling height of the heavy hammer is 0.25–2 m. According to Formula (10), the corresponding impact velocities are shown in Table 3. In the simulation, the heavy hammer is directly given an impact velocity.

The mold, upper, and lower die punching were set as rigid bodies, and the powder was a deformable body. The heavy hammer first contacted the upper die punch after falling, and the lower die punch also was required to play a load-bearing role. Therefore, materials with good toughness such as forged steel or powder metallurgy steel should be selected. The size structure of the upper and lower die punching is also important. When the ratio of the diameter of the upper die punch to the weight hammer is 1, the stress concentration is minimized. So, in the simulation mechanism, the diameter of the heavy hammer was equal to the diameter of the upper and lower die punches, with a diameter of 16 mm for the upper and lower die punches. The lower punch and the mold adopted a completely fixed constraint method, and the mold was simplified as a shell unit. The entire stamping process took a range of 2–4 ms, and the diameter of the mold cavity was set to 16 mm, with a friction coefficient of 0.2. The transient impact module in MSC Marc software was used for the solution.

### 2.4. Theoretical Analysis of Impact Process

High-speed powder compression molding is essentially a free falling motion of a heavy hammer. After falling, the heavy hammer collides with the upper mold to achieve energy transfer. At this moment of collision, an impact force *F_i_* is generated, causing the powder to form.

This paper assumes that the impact energy of heavy hammer is *E_n_*, the mass of the heavy hammer is *M*, and the mass of the upper mold punch is *m*. The initial contact velocity between the heavy hammer and the upper mold punch is *v*_1_, and then its joint motion velocity with the upper mold punch is *v*_2_. According to the kinetic energy theorem and momentum theorem, the following can be obtained:

(9) $$E_{n}=\frac{1}{2}Mv_{1}^{2}=Mgh$$

(10) $$v_{1}=\sqrt{2gh}$$

(11) $${Mv}_{1}=(M+m)v_{2}$$

(12) $$v_{2}=\frac{M}{\left(M+m\right)}v_{1}$$

We set the overall displacement of the powder as ∆S. The impact force *F_i_* can be obtained from Newton’s second law as follows:

(13) $$F_{i}=\frac{d(mv)}{dt}=\frac{dE}{dS}=\frac{\frac{({Mv_{1})}^{2}}{2(M+m)}}{∆S}=\frac{{{(Mv}_{1})}^{2}}{2(M+m)∆S}$$

## 3. Results and Discussion

### 3.1. Model Verification

**Figure 4:**
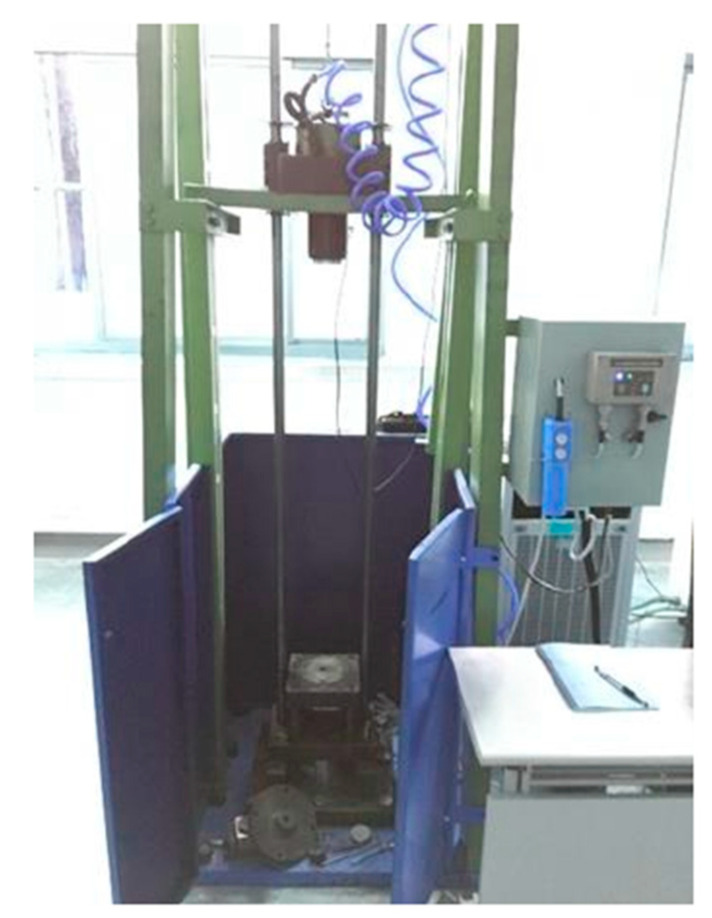
Practical picture of HVC machine [17].

**Figure 5:**
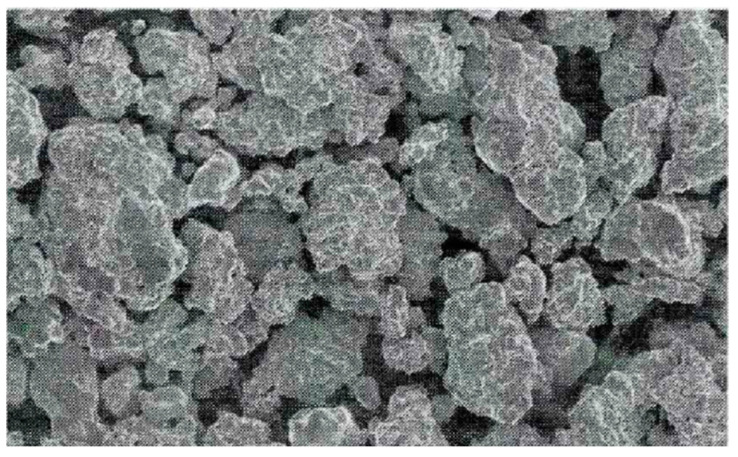
Meso-morphology of powder.

This article adopts a modeling method similar to Li Lei [6], Xi Zhong an [7], and Wang Wen chao [8]. The model size, material, boundary, and impact conditions are identical to the experiments in reference [17]. The BL-GSTZ-1 HVC device was used to carry out high-speed impact, and the device is shown in Figure 4. The meso-morphology of powder is shown in Figure 5. Other parameters of the powder are detailed in Table 1 and Table 2 and Figure 2.

**Figure 6:**
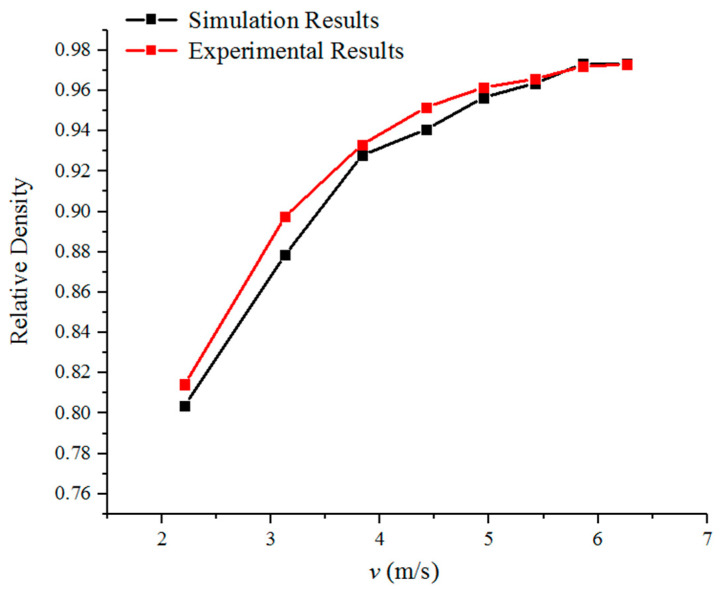
The relative density of simulation and experiment results.

The results of the numerical simulation and experimental are shown in Table 4; the corresponding comparison chart is shown in Figure 6. It can be seen that the numerical simulation and experimental results are consistent, and the numerical simulation model established in this paper can reliably simulate the HVC process of powder metallurgy.

### 3.2. The Process Analysis of HVC

In the ordinary molding process, the change in compact density can be divided into three stages. In the first stage, the powder particles completely fill the pores in the compact due to displacement, and the compact density increases linearly with pressure. In the second stage, due to the balance between external pressure and resistance during pressing, the density of the compact remains unchanged. In the third stage, the external pressure exceeds the yield strength of the powder particles, and the particles begin to undergo plastic deformation or even fracture. The density of the compact increases with the increase in external pressure, but the increasing trend slows down compared to the first stage. A metal powder with good plasticity does not exhibit a significant second stage, so the relationship curve between the density of the compact and the compression pressure is approximately parabolic.

**Figure 7:**
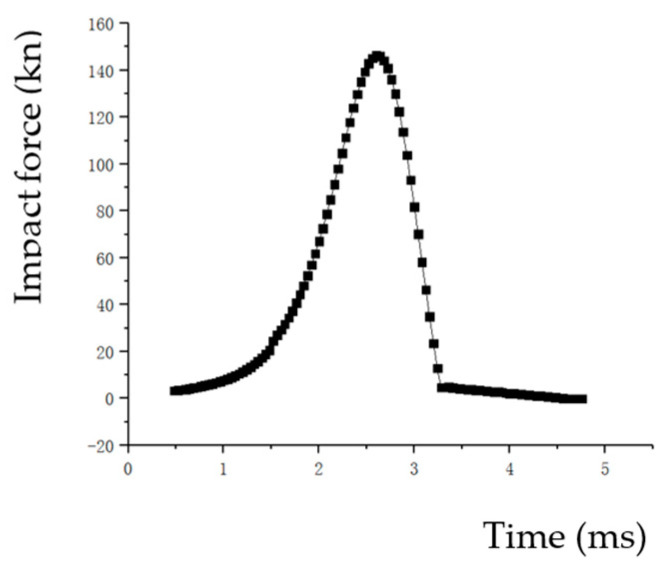
The curve of impact force and time (*v* = 3130 mm/s).

**Figure 8:**
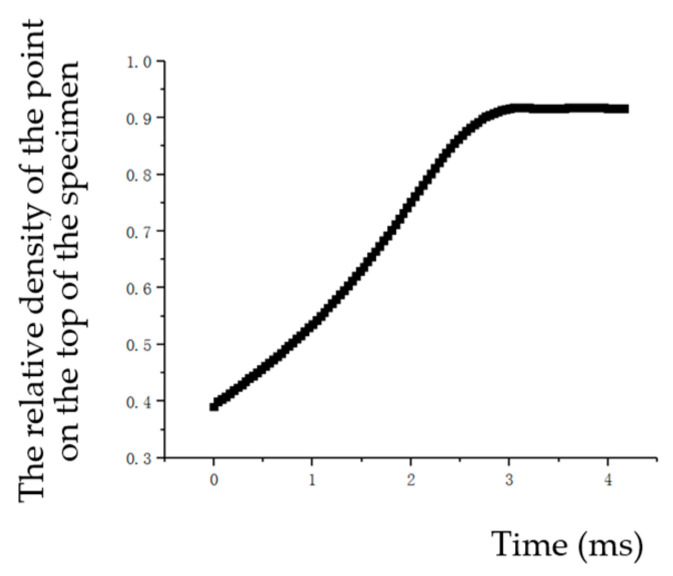
The relative density and time curve of a point on the top of the specimen (*v* = 3130 mm/s).

**Figure 9:**
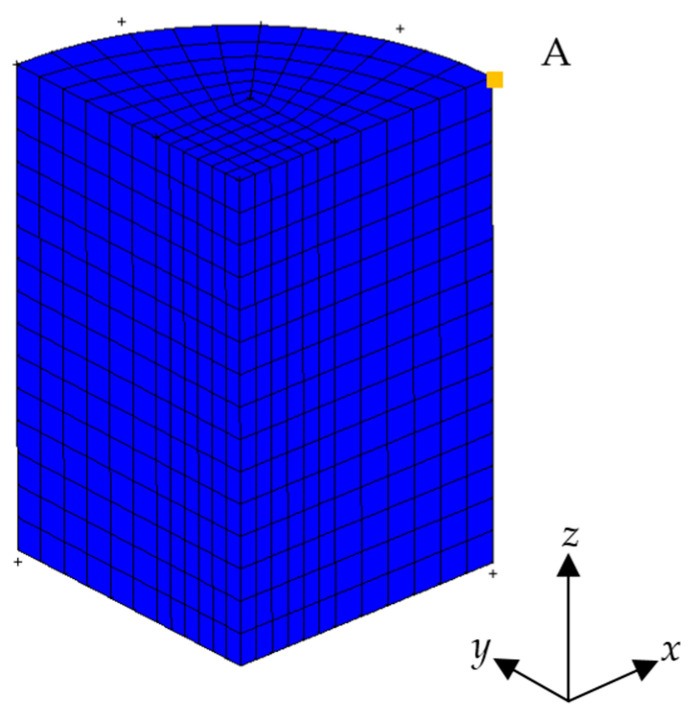
The position A of a point on the top of the powder.

**Figure 10:**
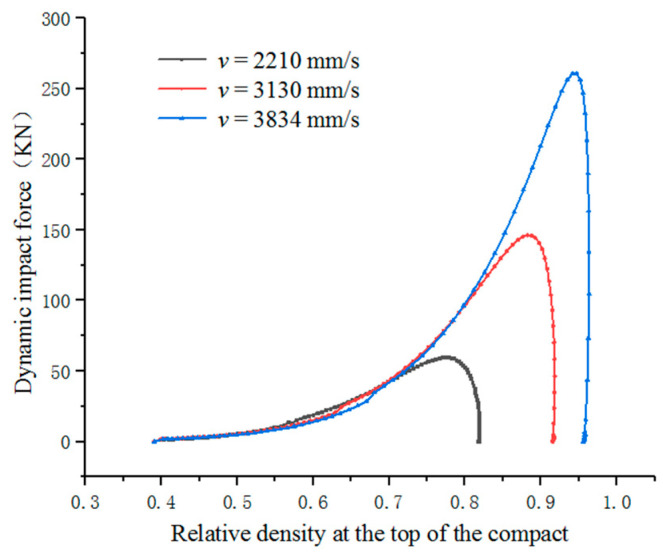
The variation in relative density with dynamic impact force at different impact velocities.

We can obtain the curve of impact force and time by software post-processing as shown in Figure 7. And we can obtain the relative density and time curve of the point A on the top of the specimen as shown in Figure 8 and Figure 9. The relative density and dynamic impact force curves for point A can be obtained by combining Figure 7 and Figure 8. The relative density and dynamic impact force curves of point A at different velocities are shown in Figure 10.

As shown in Figure 10, in the HVC process, the relative density of the compact increases rapidly with the increase in impact force in the first stage. The second stage does not appear significant, and the higher the impact velocity, the shorter the time it takes for this stage to occur, even if it is almost non-existent. In the third stage, as the impact force changes, the density of the compact slowly increases until it no longer changes.

### 3.3. Compaction Equations

The compression equation can quantitatively reflect the densification process of the compact, which has important guiding significance. This paper explores and studies the densification of the HVC process of iron-based powder metallurgy by combining the Kawakita’s powder compaction equation, Heckel compression equation, Huang Peiyun compression equation, and Balshin compression equation with simulation experimental results. The results are shown in Table 5.

#### 3.3.1. Kawakita’s Powder Compaction Equation

Kawakita’s powder compaction equation can be used for the compression molding process of metal powders, which is the relationship between the volume compression rate *C* of the powder and the compression pressure *P* [18]. Its expression is as follows:

(14) $$C=\frac{a_{1}b_{1}P}{1+b_{1}P}$$

where *C* is the volume compression rate;

*P* is the powder pressing pressure; and

*a*_1_ and *b*_1_ are parameter;

Equation (14) can be transformed into the following:

(15) $$\frac{1}{C}=a_{1}+b_{1}\frac{1}{P}$$

**Figure 11:**
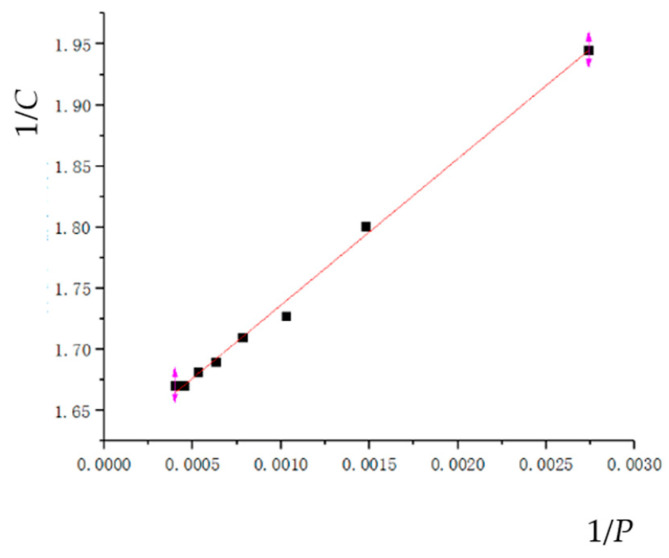
Kawakita’s powder compaction equation.

According to the simulation results, the linear equation parameters and correlation coefficients are fitted as shown in Table 6, and the fitting curve is shown in Figure 11.

#### 3.3.2. Heckel Compaction Equation

The Heckel compaction equation is as follows:

(16) $$ln\left[1/\left(1-\rho \right)\right]=a_{2}P+b_{2}$$

where ρ is the relative density;

*P* is the powder pressing pressure; 

*a*_2_ and *b*_2_ are the parameters.

**Figure 12:**
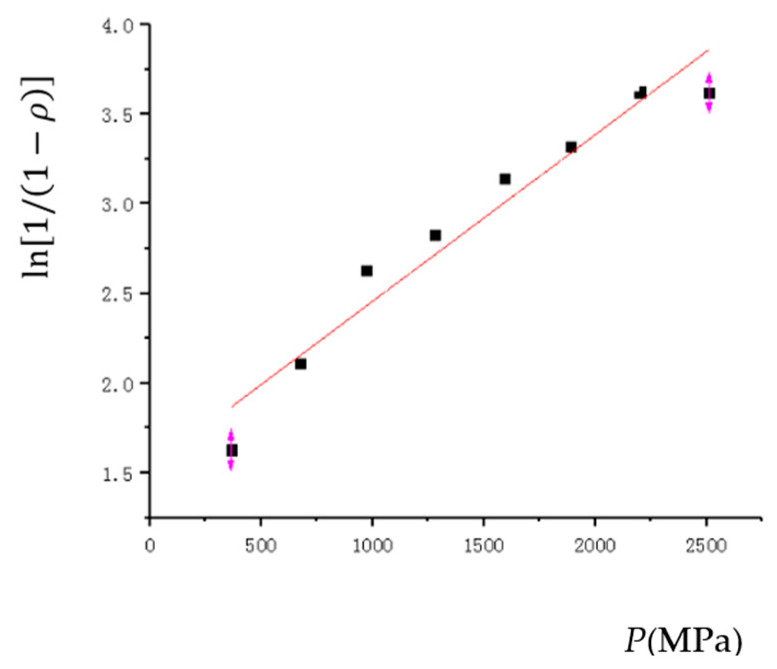
The Heckel compaction equation.

According to the simulation results, the linear equation parameters and correlation coefficients are fitted as shown in Table 6, and the fitting curve is shown in Figure 12.

#### 3.3.3. Huang Peiyun Compression Equation

Huang Peiyun analyzed the force and density during the pressing process using a double logarithmic function, and provided the corresponding formula as shown below. This theory is more suitable for molding and isostatic pressure within a certain pressure range.

(17) $$lgP=a_{3}lgln\frac{\left(\rho_{m}-\rho_{0}\right)\rho }{(\rho_{m}-\rho )\rho_{0}}+b_{3}$$

where $\rho_{m}$ is the theoretical density; $\rho_{0}$ is the initial density; ρ is the compact density;

*P* is the powder pressing pressure; and

*a*_3_ and *b*_3_ are the parameters.

**Figure 13:**
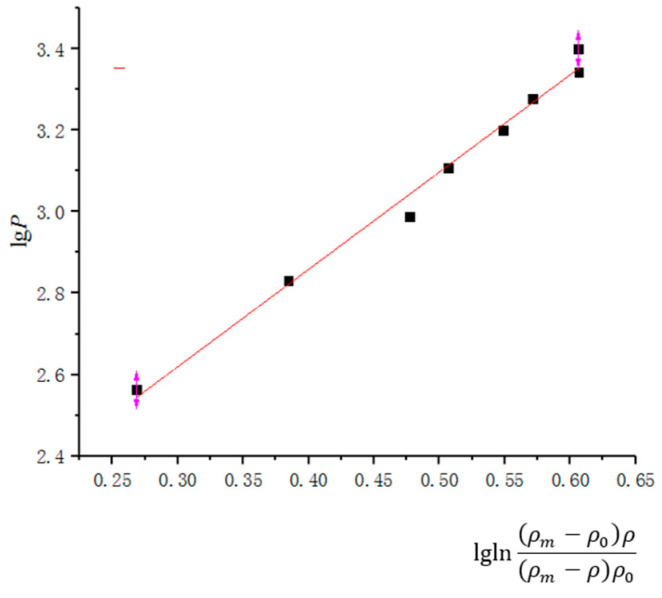
Huang Peiyun compaction equation.

According to the simulation results, the linear equation parameters and correlation coefficients are fitted as shown in Table 6, and the fitting curve is shown in Figure 13.

#### 3.3.4. Balshin Compression Equation

Balshin derived the equation between compression force and relative density through Hooke’s law. This theory is suitable for brittle and hard particles within a certain pressure range. Its expression is as follows.

(18) $$\frac{1}{\rho }=a_{4}lnP+b_{4}$$

where  ρ is the relative density;

*P* is the powder pressing pressure; and

*a*_4_ and *b*_4_ are the parameters.

**Figure 14:**
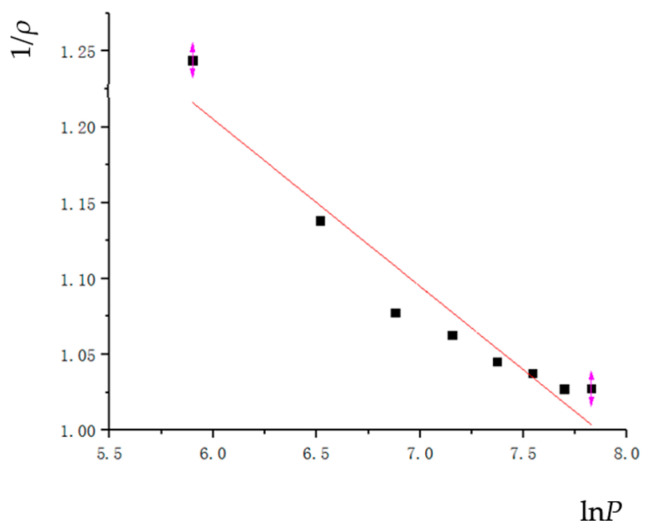
The Balshin compaction equation.

According to the simulation results, the linear equation parameters and correlation coefficients are fitted as shown in Table 6, and the fitting curve is shown in Figure 14.

#### 3.3.5. Comparison of Compaction Equations

As shown in Table 6, four commonly used classical compaction equations were fitted with empirical formulas under the HVC process, and reliability comparisons were made. After comparison, it was found that the Kawakita and Huang Peiyun compaction equations were better, with a reliability of 0.99596 and 0.98895, respectively. However, the Heckel and Balshin compaction equation fitting curves had relatively low reliability and were not in line with the actual situation of the HVC process. Through this fitting curve, it can be seen that the Kawakita compression equation is more suitable for low compression pressure and has a good fitting accuracy. The Huang Peiyun compression equation is based on the Kawakita’s compression equation, taking into account factors such as stress, strain, and work hardening. When the compression pressure is high, its fitting accuracy is also relatively good. At the same time, deviation only occurs when the compact density reaches 97% or above.

### 3.4. The Effect of Impact Velocity on the Relative Density Distribution Cloud Map

**Figure 15:**
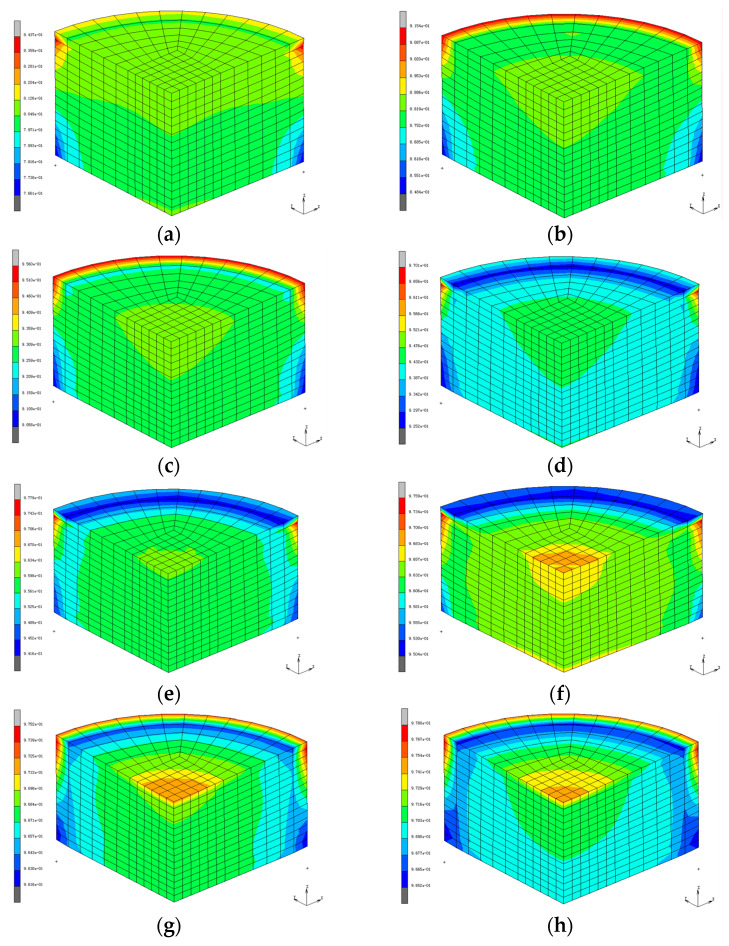
Relative density nephograms of compact under different impact speeds. (**a**) *v* = 2210 mm/s. (**b**) *v* = 3130 mm/s. (**c**) *v* = 3834 mm/s. (**d**) *v* = 4427 mm/s. (**e**) *v* = 4950 mm/s. (**f**) *v* = 5422 mm/s. (**g**) *v* = 5857 mm/s. (**h**) *v* = 6261 mm/s.

The cloud charts of relative density distribution under different impact velocities are shown in Figure 15. It can be seen from the cloud charts that the uneven density of the compact is mainly concentrated on the edges of the upper and lower end faces of the compact. The closer to the edge of the compact, the more uneven the density distribution.

According to Figure 15, the performance indexes of compact density at different impact velocities can be calculated as shown in Table 7. The standard deviation represents the uniformity of the relative density. The smaller the standard variance, the better the density uniformity. It can be seen that increasing the impact speed can effectively improve the density uniformity of the compact.

### 3.5. The Effect of Impact Energy on Relative Density

**Figure 16:**
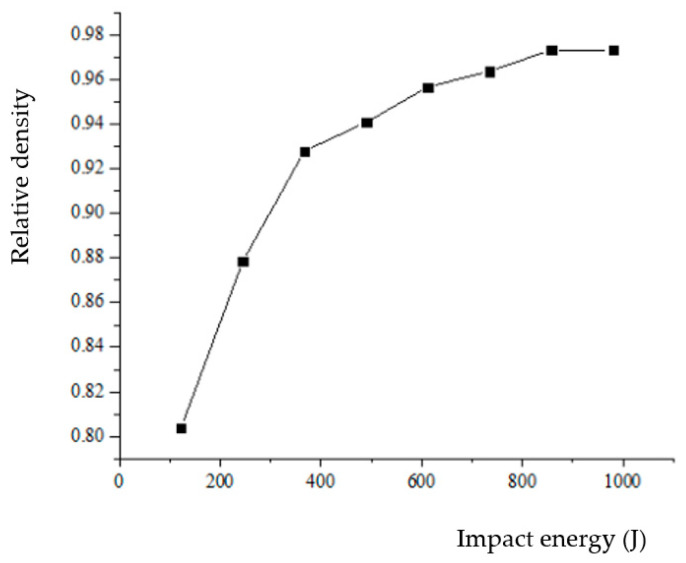
Relative density of compact under different impact energy.

In the compaction equation, the compaction pressure is for static pressure. However, for the HVC process, the pressure is a process of rapidly increasing to a maximum value and then rapidly decreasing. The force acting on the compact is a stress wave. The relationship between energy and density cannot be completely and accurately characterized by the maximum impact force or average impact force. At the same time, the compact density is also related to the mass of the hammer. Therefore, in this paper, we used the impact energy to characterize it. Through HVC simulation testing, we obtained the relationship between impact energy and the relative density of the compact, as shown in Figure 16. As can be seen from Figure 16, the greater the impact energy, the greater the relative density, but when the impact energy increases to about 600 J, the growth rate of the relative density slows down. That is to say, with the increasing impact energy, the efficiency of improving the relative density is slowing down.

## 4. Conclusions

The densification mechanism of the HVC process of iron-based powder was analyzed by FE method. The simulation results are consistent with the experimental results, proving the reliability of the model. The research method in this paper provides an effective path for the study of the HVC densification process, laying a foundation for further research on the influence of various factors on the compact density. After analyzing the high-speed pressing process, it was found that HVC process was different from ordinary molding, with almost no second deformation stage.

Meanwhile, the results were fitted with the Kawakita’s powder compaction equation, Heckel compression equation, Huang Peiyun compression equation, and Balshin compression equation. It was shown that the best fitted model was the Kawakita’s powder compaction equation, which can be expressed as $\frac{1}{C}=1.61637+119.89106\frac{1}{P}$. Furthermore, we studied the relative density distributions at different velocities and impact energies. It was shown that the uneven density of the compact is mainly concentrated on the edges of the upper and lower end faces of the compact, and the higher the velocity, the more uniform the density distribution. Finally, the effect of impact energy on relative density was studied. It can be concluded that the efficiency of improving the relative density is slowing down with the increasing impact energy.

In general, although we have analyzed the densification process of iron-based powder with a cylinder shape in the paper, it can be proved that the FEM model can be applied in simulating the HVC process with a complicated geometry and composite powders, which is widely used in practical manufacturing.

## Figures and Tables

**Table 1:** The composition of Distaloy AE iron-based powder.

Composition	Ni	Cu	Mo	C	Fe
Content (percentage by weight)	4.01	1.48	0.49	0.09	126.289

**Table 2:** Initial conditions and constraints.

The Mass of Heavy Hammer (Kg)	The Parameters of the Initial State Specimen			
	Mass (g)	Diameter *D* (mm)	Initial Loose Height *H* (mm)	Initial Relative Density
50	8	16	12.96	0.3906

**Table 3:** The falling height and the corresponding impact velocities of the heavy hammer.

Name	Value							
The falling heights of heavy hammer (m)	0.25	0.5	0.75	1	1.25	1.5	1.75	2
Impact velocities *v* (m/s)	2.214	3.13	3.834	4.427	4.95	5.422	5.857	6.261

**Table 4:** The results of numerical simulation and experimental research under different impact velocities.

Impact Velocity (m/s)	Relative Density (Experiment)	Relative Density (Simulation)	Impact Force *F_i_* (KN)
2.214	0.8144	0.803826045	18.181
3.130	0.8976	0.878552517	33.459
3.834	0.9333	0.927914031	48.739
4.427	0.9518	0.940879169	64.131
4.950	0.9616	0.956675087	79.553
5.422	0.9658	0.963747452	95.201
5.857	0.9721	0.973309341	110.660
6.261	0.9730	0.973193686	126.289

**Table 5:** Simulation results.

Impact Velocity (mm/s)	Initial Volume	Compact Volume	Volume Compression Rate	Impact Stress (MPa)
2214	6.50 × 10^2^	315.6487	5.14 × 10^−1^	365
3130	6.50 × 10^2^	288.8008	5.55 × 10^−1^	676
3834	6.50 × 10^2^	273.4377	5.79 × 10^−1^	973
4427	6.50 × 10^2^	269.6698	5.85 × 10^−1^	1280
4950	6.50 × 10^2^	265.2172	5.92 × 10^−1^	1590
5422	6.50 × 10^2^	263.2709	5.95 × 10^−1^	1890
5857	6.50 × 10^2^	260.6845	5.99 × 10^−1^	2200
6261	6.50 × 10^2^	260.7155	5.99 × 10^−1^	2510

**Table 6:** Empirical formula for compaction equation.

Name	Compaction Equation	Parameter *a*	Parameter *b*	Reliability Characterization Coefficient R^2^
Kawakita	$\frac{1}{C}=a_{1}+b_{1}\frac{1}{P}$	1.61637	119.89106	0.99596
Heckel	$ln\left[1/\left(1-\rho \right)\right]=a_{2}P+b_{2}$	9.28826 × 10^-4^	1.52841	0.94687
Huang Peiyun	$lgP=a_{3}lgln\frac{\left(\rho_{m}-\rho_{0}\right)\rho }{(\rho_{m}-\rho )\rho_{0}}+b_{3}$	2.38947	1.90307	0.98895
Balshin	$\frac{1}{\rho }=a_{4}lnP+b_{4}$	−0.11022	1.86642	0.92916

**Table 7:** Relative density index of compacts at different impact velocities.

Impact Velocity (mm/s)	Minimum Relative Density	Maximum Relative Density	Average Relative Density	Standard Deviation	Uniformity
2214	0.7661	0.8437	0.8049	0.0388	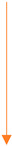
3130	0.8484	0.9154	0.8819	0.0335	
3834	0.9059	0.959	0.93245	0.02655	
4427	0.9252	0.9701	0.94765	0.02245	
4950	0.9416	0.9779	0.95975	0.01815	
5422	0.9504	0.9759	0.96315	0.01275	
5857	0.9616	0.9752	0.9684	0.0068	
6261	0.9652	0.978	0.9716	0.0064	better

## Data Availability

Data are contained within the article.

## Nomenclature

HVC	High Velocity Compression
*F*	The Yield Strength
*E*	The Elastic Modulus
ρ	The Relative Density
*υ*	Poisson’s Ratio
*E_n_*	Impact Energy of Heavy Hammer
*F_i_*	The Impact Force
*C*	The Volume Compression Rate
*P*	The Compression Pressure
